# Intra-tumoural vessel area estimated by expression of epidermal growth factor-like domain 7 and microRNA-126 in primary tumours and metastases of patients with colorectal cancer: a descriptive study

**DOI:** 10.1186/s12967-014-0359-y

**Published:** 2015-01-16

**Authors:** Torben Frøstrup Hansen, Boye Schnack Nielsen, Anders Jakobsen, Flemming Brandt Sørensen

**Affiliations:** Department of Oncology, Vejle Hospital, part of Lillebaelt Hospital, Kabbeltoft 25, 7100 Vejle, Denmark; Bioneer A/S, Hørsholm, Denmark; Department of Clinical Pathology, Vejle Hospital, part of Lillebaelt Hospital, Vejle, Denmark; Institute of Regional Health Research, University of Southern Denmark, Region of Southern Denmark, Denmark

**Keywords:** Angiogenesis, Colorectal neoplasms, Epidermal growth factor like domain 7, Metastases, microRNA-126

## Abstract

**Background:**

Understanding the biological properties of potential drug targets are important. This is especially true for anti-angiogenic therapies in the search for potential biomarkers. The aim of the present descriptive study was to analyse the intra-tumoural expressions of epidermal growth factor-like domain 7 (EGFL7) and microRNA-126 (miRNA-126) in primary tumours from patients with stage II-IV colorectal cancer (CRC) and in paired samples of primary tumours, regional lymph node metastases and distant metastases.

**Methods:**

A total of 126 patients were included. Analyses were performed on resections of primary tumours, regional lymph node metastases, and large needle biopsies from distant metastases. EGFL7 was analysed by immunohistochemistry (IHC) and miRNA-126 by *in situ* hybridization (ISH). Both biomarkers were quantified by image guided analyses to determine the relative fraction estimates of vessel areas (VA).

**Results:**

The intra-tumoural EGFL7 VA was significantly higher in primary tumours from patients with recurrent disease than in patients without relapse in both stage II and III, p = 0.019 and p = 0.001, respectively. The EGFL7 VA was significantly higher and the miRNA-126 VA significantly lower in regional lymph node metastases compared to primary tumours, p = 0.01 and p < 10^−6^, respectively. Furthermore, the miRNA-126 VA in liver metastases was significantly lower than in the primary tumours, p = 0.02.

**Conclusion:**

The intra-tumoural expression of EGFL7 in early stages of CRC may influence the risk of post-surgical recurrence. Differential expression of miRNA-126 seems more pronounced in disseminated disease, which supports its role as a regulator in the metastatic process.

**Electronic supplementary material:**

The online version of this article (doi:10.1186/s12967-014-0359-y) contains supplementary material, which is available to authorized users.

## Background

Targeting angiogenesis is a well established strategy in the treatment of patients with metastatic colorectal cancer (mCRC) and the clinical benefit is well documented [1,2]. The initial experiences were based on an antibody targeting the vascular endothelial growth factor A (VEGF-A), one of the most important growth factors in the onset of the angiogenic process [3,4]. The clinical benefit, however, of targeting VEGF-A is rather limited and may be restricted to a subgroup of patients, and new strategies for controlling tumour angiogenesis are constantly being evaluated.

One strategy, currently under investigation in clinical trials, is the targeting of another important pro-angiogenic protein, the epidermal growth factor-like domain 7 (EGFL7). The *EGFL7* gene was identified around 2003 and the gene product was characterised as a protein restricted to the vascular system and highly vascularised tissues [5-7]. The expression of EGFL7 is up-regulated during pathophysiological angiogenesis [8], where it is secreted to the extracellular matrix (ECM), and guides the vascular sprouting process [9]. EGFL7 is important for tubulogenesis [6] and is able to inhibit endothelial cell (EC) adhesion molecules causing blood vessels to become leaky [10]. In human cancers elevated levels of EGFL7, assessed by immunohistochemistry (IHC) as well as qPCR, have been associated with increased risk of metastatic spread in several solid tumours [10-13]. However, the evidence on EGFL7 in colorectal cancer (CRC) is still very sparse [11,14].

Another therapeutic strategy that may become available in the near future is the targeting of microRNA (miRNA). In the context of anti-angiogenic therapy miRNA-126 replacement may constitute an exiting approach. The miRNA-126 is a highly EC specific miRNA [15,16] regulating EC proliferation, migration, and survival by modulating VEGF-A and angiopoietin 1 driven signalling cascades [17-20]. Regulating blood vessel integrity and extravasation of inflammatory cells into the tumour compartment constitute other functions regulated by this miRNA [17,18,21]. MicroRNA-126 is encoded by intron 7 of the *EGFL7* gene [18], and the EGFL7 mRNA is suggested to constitute one of its many targets [22], adding complexity to the interplay of this angiogenic couple. The clinical evidence on miRNA-126 in CRC argues for a deregulated expression in tumour tissue [23,24] and a possible clinical importance at both early and advanced stages of the disease [11,14,25,26].

We have previously shown that high miRNA-126 expression in primary tumours, determined by *in situ* hybridization (ISH) analysis, is predictive of response to chemotherapy in mCRC [25], and that this association is related to miRNA-126 and not the general vascular density in the tumours [27], indicating that the miRNA-126 related vascularisation of primary tumours is an important predictor of response to therapeutic intervention.

Translational studies in mCRC are often based on analyses of the resected primary tumour. Whether these results can be extrapolated to the metastatic lesions that are targeted by the treatment, is often unknown. Analysing putative biomarkers in paired samples of metastases and primary tumours may thus provide useful biological insight. Furthermore, analysing biomarker expression at earlier disease stages may likewise be of importance, when evaluating the therapeutic potential of the given pathway in an adjuvant setting.

The aim of this descriptive study was to analyse the expression of EGFL7 and miRNA-126 in the primary tumours of stage II-IV CRC and whether this expression changed in paired samples of primary tumours, regional lymph node metastases and distant metastases.

## Methods

Reporting in this study is in accordance with the BRISQUE criteria [28].

### Patient characteristics

**Figure 1:**
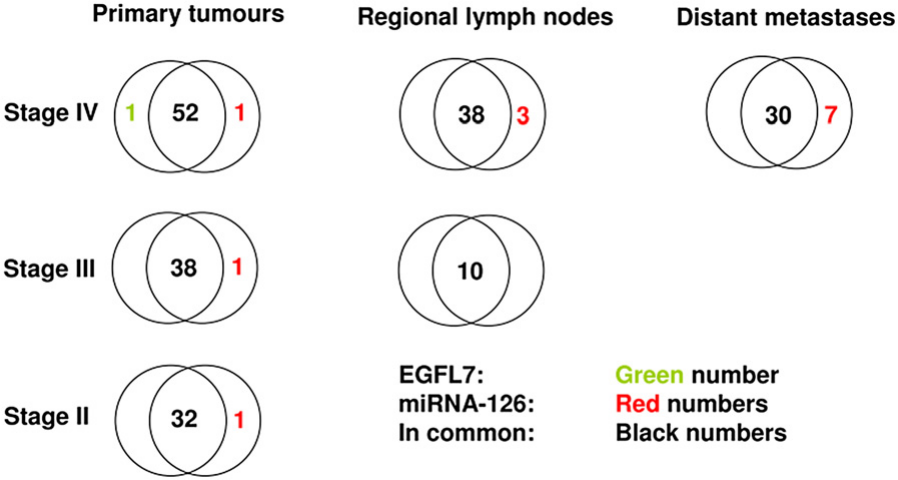
**Sample distributions according to location and analysed parameters.** (EGFL7: Epidermal growth factor-like domain 7; miRNA-126: microRNA-126).

This biology focused, descriptive study included patients with histologically verified CRC stage II-IV. Patients were selected according to the following criteria: Patients with stage II or III disease followed for at least 6 years after surgery without any sign of relapse, patients with stage II or III disease with histologically verified relapse at distant organ sites within 6 years after surgery, and patients with primary disseminated disease (stage IV). A total of 126 patients were retrospectively included. All patients had their primary tumour surgically removed. Regional lymph node metastases and distant metastases (synchronous and metachronous) were collected if available (Figure 1). Patients were operated at six different centres in Denmark between June 1998 and July 2012.

The study was approved by the Regional Scientific Ethical Committee and the Danish Data Protection Agency (20010078, S-20080104, S-20100005, VF-20060115, 2007-41-0252), and written informed consent was obtained from all patients enrolled in the study.

### Sampling

Available paired bio-specimens consisted of resected primary tumours and regional lymph node metastases, and large needle biopsies from distant metastases as shown in Figure 1. Some of the bio-specimens contained limited tumour tissue allowing only the EGFL7 or miRNA-126 analysis.

All histological samples followed routine formaldehyde fixation and paraffin embedding (FFPE) and were stored and transported at room temperature. The median storage time from sampling to analysis was 5.7 years. All FFPE tissue blocks were processed at the Department of Pathology, Vejle Hospital, Denmark. Tissue sections for EGFL7 IHC and miRNA-126 ISH analyses were cut adjacently from the same FFPE tissue block.

### EGFL7 immunostaining

Tissue sections were stained with an anti-EGFL7 antibody as previously described [14]. Tissue sections, 4 μm thick, were mounted on coated slides and dried for half an hour at 60°C. Deparaffinisation was performed in estisol for 10 min at room temperature. Rehydration was performed in graded alcohol solutions (99-70%). Blocking of endogenous peroxidase was achieved by incubation in 3% hydrogen peroxide for 5 min. Antigen demasking was done by heat-induced epitope retrieval in a microwave oven, using a TEG buffer (TRIS 10 mM, EGTA 0.5 mM, Titriplex®-VI) at pH 9 for 10 min at 1000 W and 15 min. at 440 W. Tris-buffered saline (TBS)/Tween pH 7.6 was added for 5 min. after cooling at room temperature. The anti-EGFL7 antibody was a rabbit polyclonal antibody (ab115786, Abcam Cambridge, UK) used in a 1:200 dilution (diluted in Dako REAL Antibody Diluent, S2022) and incubated for 90 min. (room temp.). After washing in TBS/Tween the visualisation was accomplished using Dako’s EnVision + System-HRP (DAB) for Rabbit Primary Antidodies™, K4011 for 30 min. All sections were counterstained with Mayer haematoxylin.

The specificity of the anti-EGFL7 antibody was tested by pre-incubation of the antibody with recombinant EGFL7 protein. 4 μl recombinant EGFL7 (Novus Biologicals H00051162-P01) and 1 μl of the anti-EGFL7 antibody were mixed in 200 μl dilution buffer (Dako EnVision FLEX WASH BUFFER K8007) to give approximately 2.4 μM antigen versus 0.6 μM antibody. The solution was left for 30 min at room temperature followed by 90 min incubation on the tissue sections. This resulted in greatly reduced staining of ECs and abolished staining of smooth muscle cells, adipocytes, tumour cells as well as enteric neuronal cells and weak background staining. These observations suggest that the antibody recognise EGFL7 in EC, and probably also one or more other similar proteins (Additional file 1: Figure S1).

### MicroRNA-126 in situ hybridization

The miRNA-126 ISH was performed essentially as previously described [29,30]. In brief, 6-μm thick tissue sections were pre-treated with a proteinase-K solution followed by hybridization with a double 6-carboxyfluorescein (FAM)-labelled Locked Nucleic Acid (LNA) miRCURY probe (LNA™ microRNA detection probe, Exiqon A/S, Denmark). After stringent washes in SSC buffers, the probes were detected with alkaline phosphatise-conjugated sheep anti-FAM Fab fragments (Roche). The 4-nitro-blue tetrazolium (NBT) and 5-brom-4-chloro-3′-Indolyl-phosphate (BCIP) substrate (Roche) were added and samples were incubated leading to a blue precipitate (the ISH signal). Counterstaining was performed with nuclear fast red. All slides were processed in a Tecan Freedom Evo automated hybridization instrument (Tecan, Männedorf, Switzerland) [30] in series of 48 slides per run.

### EGFL7 and microRNA-126 image analyses

Image acquisition and analysis was carried out according to the methodology previously described [14].

*EGFL7*: Image analysis was performed using the Visiopharm integrated microscope and software module (Visiopharm, Hørsholm, Denmark). Whole slide images were obtained for each slide using x1.5 objective. The tumour area was encircled in order to obtain random, systematic sampling of x20 images (x282 total magnification on the computer monitor) for quantification of the EGFL7 expression. Two image acquisition configurations were applied depending on whether the samples were whole tumour resections, lymph nodes or large needle biopsies. For tumour resections, up to 25 non-overlapping x20 images per sample were collected. All images were *within* the tumour tissue compartment. For large needle biopsies, x20 images were collected to cover the *whole* tissue area. A fraction of the images contained limited tumour tissue compartments. The pixel classifier prepared for the tumour resections generated estimates of the EGFL7 expression that were 11% higher than those obtained from the large needle biopsy specific classifier, probably due to increased area contribution from empty glands associated with smaller biopsies. A factor 1.11 was consequently used to correct for these estimates. To allow direct comparison between the estimates of EGFL7 expression from resection samples and the biopsy samples, all estimates were calculated as [[area of stained ECs]*[1.11]]/[total tissue area detected]. The total tissue area detected also included nuclei and connective tissue, as mean values of x20 images for each patient. Thus, the estimates of EGFL7 expression are presented as vessel area fractions (VA) without a dimension. Strong DAB staining was only seen in EC while faint and sporadic DAB staining was detected in some other cell types that resulted in a minor contribution to the EGFL7 estimate. The threshold of the classifier was thus adjusted to count only the strong DAB signal resulting in an estimate virtually specific for EC expression of EGFL7. The staining of the large needle biopsies resulted in expression of more widespread background levels in a few samples. Images with tumour ulceration and necrotic tissue, staining artefacts, and those with no cancer cells were excluded.

*MicroRNA-126*: Image acquisition and analysis was achieved using the same principles as described for EGFL7 with a few exceptions. The colour classifier was prepared to determine the area fraction represented by the blue miRNA-126 ISH signal. As for the EGFL7, only the strong ISH signals were included in the quantitative estimates and the mean score of VA fractions from the sampled images, and calculated for each patient as described for EGFL7. The image analysis protocols were performed by one observer (BSN) unaware of the clinical setting.

### Statistics

Median values were compared using the Wilcoxon rank sum test. Fisher’s exact test was used for comparison between categorical parameters, and linear regression analysis was used to investigate the linear association between continuous variables. All statistical calculations were carried out using the NCSS statistical software (NCSS Statistical Software, Kaysville, UT 84037, USA, version 2007). P values < 0.05 were considered significant, and all tests were two-sided.

## Results

**Figure 2:**
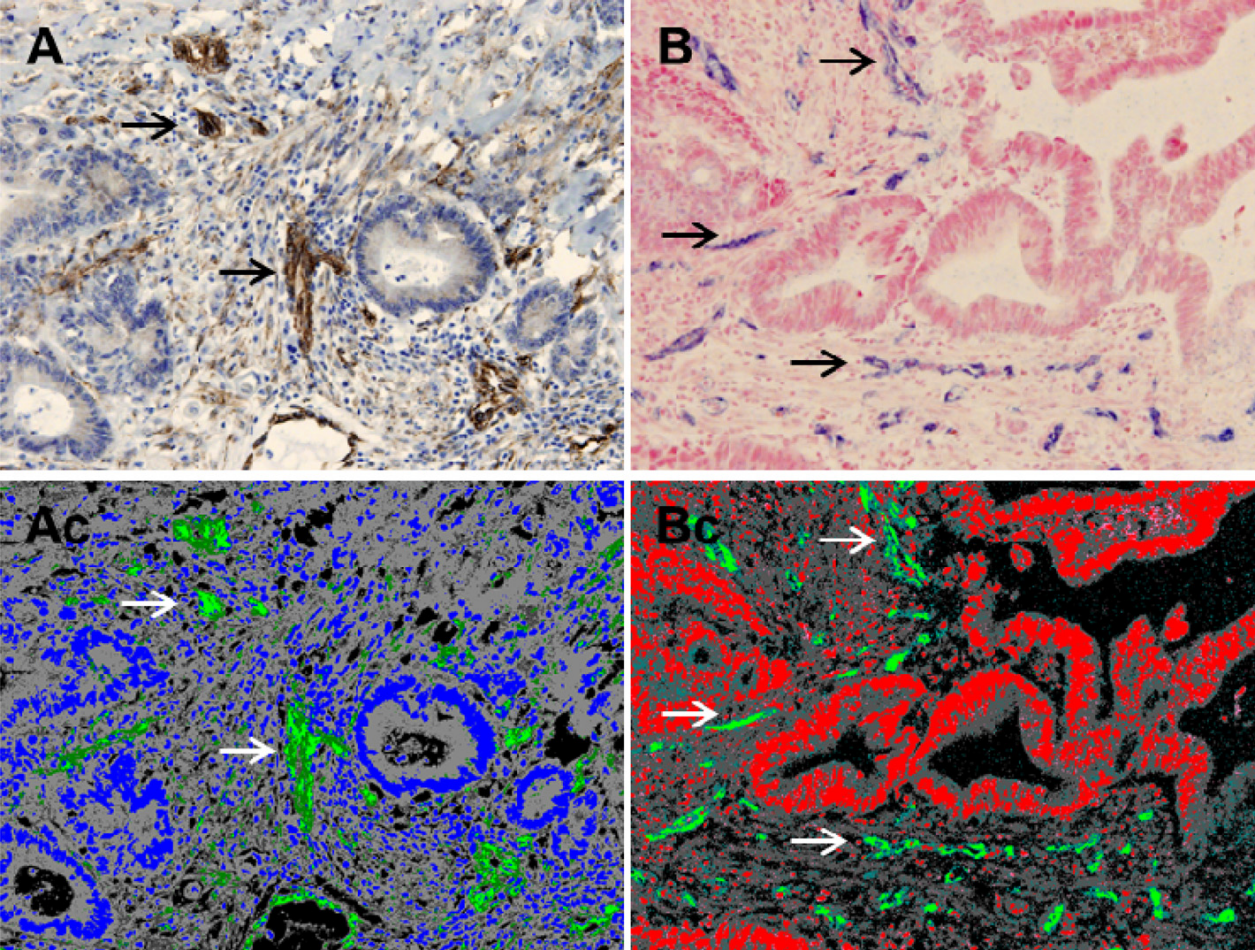
**Visualisation of EGFL7 and miRNA-126.** Representative images of **(A)**: the intra-tumoural expression of Epidermal Growth Factor-like Domain 7 (EGFL7), by immunohistochemical analysis, showing staining of endothelial cells (ECs, brown) and the corresponding classified image **(Ac)** demonstrating intense staining of ECs (green) and faint staining (dark green) of myofibroblasts. Only the intense signal was included in the quantitative estimate. **(B)** The microRNA-126 (miRNA-126) *in situ* hybridization (ISH) analysis, showing merely exclusive intra-tumoural expression of ECs (blue) and the corresponding classified image **(Bc)** demonstrating the ECs included in the quantitative estimate (green).

The intra-tumoural expressions of EGFL7- and miRNA-126 were successfully visualised in all tissue samples. Representative images of the EGFL7 immunoperoxidase staining and the miRNA-126 ISH signal and their corresponding classified images used to obtain quantitative estimates of VA are demonstrated in Figure 2. The EGFL7 immunoperoxidase resulted in intense staining of ECs (cytoplasm and cell membrane), and rather faint signals in smooth muscle cells, adipocytes, enteric neurons and sometimes cancer cells (Figure 2A). The miRNA-126 probe resulted in an intense ISH signal in blood vessels and weaker staining in other tissue structures, which was considered unspecific (Figure 2B).

### Patients

Patient characteristics according to stage and relapse are shown in Table 1. Though pre-selected for post surgical relapse or 6-year relapse free survival, patients with stage II-III disease were comparable regarding the standard clinico-pathological characteristics (p > 0.25).

**Table 1:** **Clinico-pathologic characteristics according to disease stage and relapse (N = 126)**

**Parameter**	**Stage of disease at time of operation**				
	**Stage II**		**Stage III**		**Stage IV**
	**Relapse free**	**Relapsed**	**Relapse free**	**Relapsed**	
	**N = 20 (%)**	**N = 13 (%)**	**N = 20 (%)**	**N = 19 (%)**	**N = 54 (%)**
**Gender**					
Male	12 (60)	7 (54)	9 (45)	10 (53)	22 (41)
Female	8 (40)	6 (46)	11 (55)	9 (47)	32 (59)
**Age**					
Median	70	65	66	66	64
Range	54-84	44-76	48-87	45-73	25-79
**Location**					
Colon	12 (60)	11 (85)	14 (70)	12 (63)	45 (83)
Rectum	8 (40)	2 (15)	6 (30)	7 (37)	9 (17)
**T category**					
1-3	19 (95)	12 (92)	18 (90)	16 (84)	27 (50)
4	1 (5)	1 (8)	2 (10)	3 (16)	27 (50)
**Malignancy grade** ^**a**^					
High	3 (15)	2 (18)	5 (25)	2 (18)	12 (26)
Low + medium	17 (85)	9 (82)	15 (75)	9 (82)	34 (74)
**Vascular invasion** ^**a**^					
Yes	2 (11)	2 (22)	2 (11)	1 (13)	24 (55)
No	16 (89)	7 (78)	17 (89)	7 (88)	20 (45)
**Neuronal invasion** ^**a**^					
Yes	1 (10)	1 (11)	4 (20)	1 (13)	14 (42)
No	9 (90)	8 (89)	16 (80)	7 (88)	19 (58)
**EGFL7 VA** ^**b**^					
Median (95% CI)	2.9 (1.9-4.9)	5.0 (3.0-9.0)	3.0 (1.4-4.1)	6.0 (4.0-9.0)	7.0 (4.0-9.0)
**miRNA-1276 VA**					
Median (95% CI)	6.7 (5.6-9.9)	4.3 (4.0-8.9)	6.0 (4.0-8.0)	6.9 (4.8-11.8)	8.6 (5.3-10.8)

CI: Confidence interval.

^a^Not assessed for all patients.

^b^Estimates of vessel area fraction.

Not all numbers in the parentheses equal 100 due to rounding of data.

The median, intra-tumoural EGFL7 VA was significantly higher in patients with relapsing disease compared to corresponding patients with stage II (p = 0.02) and stage III (p = 0.001), without relapse.

### Primary tumours

The median estimates of intra-tumoural EGFL7 and miRNA-126 VA in primary tumours according to stage and relapse are shown in Table 1. The median EGFL7 VA was significantly higher in patients with recurrent disease compared to patients with stage II (p = 0.02) and stage III (p = 0.001), without relapse. There was no significant difference in the median EGFL7 VA of the primary tumours between patients with nonrecurring stage II and III, or between patients with recurring stage II or III and stage IV. There were no significant differences in median miRNA-126 VA between the patient groups.

**Figure 3:**
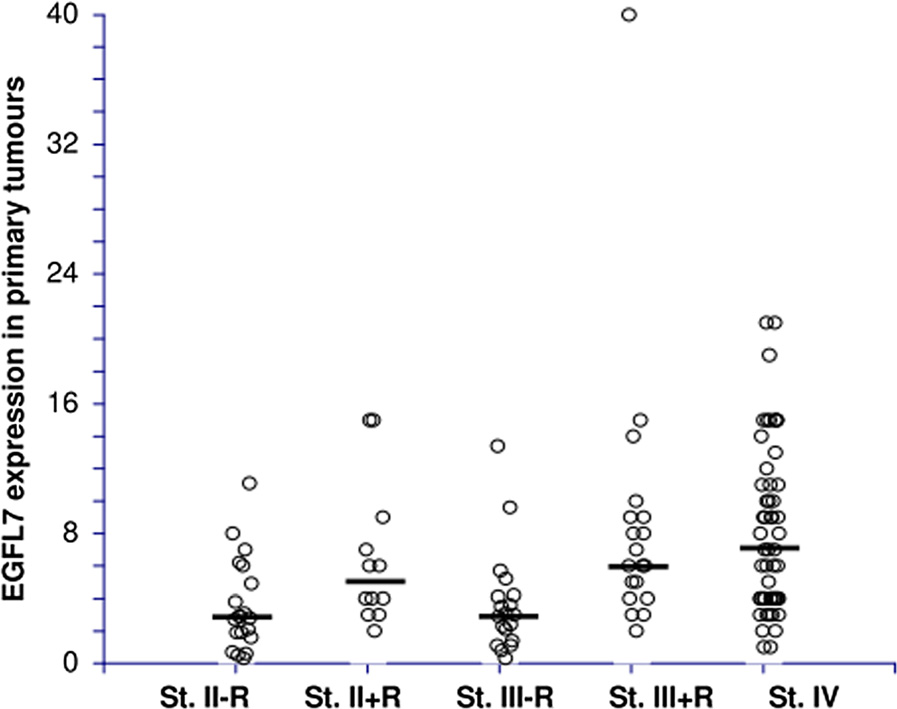
**Dot plot illustrating the intra-tumoural expressions of Epidermal Growth Factor-like Domain 7 (EGFL7) in primary tumours according to stage (St.) and relapse free (−R) and relapsed (+R) patients, respectively (N = 123).** See Table 2 for statistical differences between the medians (represented by black bars).

The distributions of EGFL7 VA according to disease stage and clinical course are shown in Figure 3.

Significant correlations between VA estimates of EGFL7 and miRNA-126 were demonstrated in patients with stage III disease, relapsed or without relapse, and stage IV, respectively (Additional file 2: Figure S2 A-E).

### Regional lymph node metastases

The median of EGFL7 and miRNA-126 VA estimates in paired samples of primary tumours and regional lymph node metastases are shown in Table 2. The differences between expressions in primary tumours and regional lymph node metastases were significant regarding both parameters with the EGFL7 demonstrating higher VA in the regional lymph node metastases and the miRNA-126 lower VA, compared to the primary tumours, respectively.

**Table 2:** **Epidermal growth factor-like domain 7 (EGFL7) and microRNA-126 (miRNA-126) expressions in primary tumours and associated lymph node/distant metastases**

**Parameter**	**EGFL7 vessel area**				**miRNA-126 vessel area**			
	**Number**	**Median**	**CI**	**p-value**	**Number**	**Median**	**CI**	**p-value**
Primary tumours	48	7.0	(4.0-9.0)	**0.01**	51	8.6	(5.3-10.8)	**<10** ^**−6**^
Lymph nodes	48	9.0	(7.0-13.0)		51	2.7	(2.1-3.4)	
Primary tumours	12	5.5	(4.0-15.0)	0.84	13	11.4	(4.8-16.1)	**0.02**
Liver metastases	12	6.7	(2.0-15.0)		13	5.1	(2.6-7.3)	
Lymph nodes	6	12.5	(2.0-21.0)	0.83	6	2.2	(0.1-4.5)	0.06
Liver metastases	6	9.8	(0.5-25)		6	6.3	(2.6-8.0)	

CI: Confidence interval.

Numbers differ in the comparisons depending on the availability of paired samples. Bold data indicates significant p-values.

A borderline significant correlation was detected between estimates of EGFL7 and miRNA-126 VA in the regional lymph node metastases (Additional file 2: Figure S2 F). No significant correlations were detected between the VA of EGFL7 and miRNA-126 in primary tumours and regional lymph node metastases, r = 0.15, p = 0.32, and r = 0.17, p = 0.24, respectively.

### Distant metastases

The median of EGFL7 and miRNA-126 VA estimates according to location of distant metastases are shown in Additional file 3: Table S1. The median miRNA-126 VA in lung metastases was significantly lower than in peritoneal metastases (p = 0.047).

For statistical power, only the VA estimates of EGFL7 and miRNA-126 in the liver metastases were compared with the corresponding expressions in the regional lymph node metastases and the primary tumours. The median miRNA-126 VA was significantly lower in liver metastases than in the primary tumours, p = 0.02 (Table 2). Furthermore, a positive significant correlation between estimates of EGFL7 and miRNA-126 VA was detected in the liver metastases, r = 0.55, p = 0.02 (Additional file 2: Figure S2 G). No significant correlations were found comparing estimates of EGFL7 and miRNA-126 VA in primary tumours and liver metastases, r = −0.26, p = 0.41, and r = −0.01, p = 0.97, respectively.

## Discussion

In the present descriptive study we describe the expression of EC-associated EGFL7 and miRNA-126 by estimating the vessel area fractions in primary tumours of different stages and in paired samples of primary tumours, regional lymph node metastases, and distant metastases from patients with CRC. The results may suggest a possible clinical importance of EGFL7 at early disease stages and a coordinated regulation of this angiogenic couple in localised as well as metastatic disease.

From the methodological point of view one may question whether the obtained VA estimates of EGFL7 and miRNA-126 in the present study are really different from traditional estimates of *e.g.* microvessel density (MVD) as obtained by CD31 and/or CD34 IHC. However, the MVD estimate is often based on hot-spot sampling, whereas the methodological approach in the present study is based on random, systematic sampling quantifying the intra-tumoural expressions of EGLF7 and miRNA-126 within the whole tumour area. In addition, being area fractions, the VAs do not represent numerical densities, but are relative fraction estimates for the individual tumours. Both CD31 and CD34 play a role in cellular adhesion and angiogenesis, but can often be detected in mesenchymal and bone marrow-derived cells in addition to the ECs, which may contribute to bias in MVD estimates. Our image analysis and sampling approach, based on intensity thresholds, diminished the contribution from mesenchymal cells to the VA estimates. On the other hand, EGLF7 is important for vascular tubulogenesis [6] and permeability [10]. Moreover, being highly specific for ECs [15,16], miRNA-126 participates in regulating EC proliferation, migration, and survival [17-20], and takes part in adjusting blood vessel integrity and thus the extravasation of inflammatory cells into the tumour compartment [17,18,21]. Thus, a range of functional aspects are associated with EGLF7 and miRNA-126, which are unrelated to that of CD31 and/or CD34. We have previously reported that MVD estimates of CD34- correlate with that of miRNA-126- [27], but the association between the parameters was weak.

The EGFL7 immunoperoxidase staining showed an intense staining of EC, but also faint signals in other cell populations, including adipocytes, smooth muscle cells, cancer cells and enteric neuronal cells. This is surprising since miRNA-126 is located within the EGFL7 precursor transcript and therefore high concordance in the expression patterns between the two is to be expected. However, it should be noted that expression of EGFL7 has been reported by others in cancer cells [12,13] and neurons [31]. Importantly, however, it was clear that the strongest EGFL7 signal was seen in ECs and the implementation of a signal intensity threshold in the image analysis, and restricting quantification within the tumour area, enabled us to obtain estimates of tumour-derived blood vessel specific EGFL7 expression. The miRNA-126 ISH signal was generally EC specific in both the primary tumours and metastatic lesions, in accordance with our previous findings [27]. Our observation that EGFL7 immunostaining was seen in non-EC cells questions the specificity of the EGFL7 antibody employed. Our antibody specificity test suggested that the antibody specifically reacted with the other cell populations, including adipocytes, smooth muscle cells, and cancer cells. If EGFL7 expression is EC specific, this observation implies that the antibody cross-reacts with one or more similar proteins expressed in those cell populations. On the other hand, if the EGFL7 immunostaining observed in the non-EC compartments represents genuine EGFL7, the different expression patterns between EGFL7 and miRNA-126 suggest differential post-transcriptional processing involving parallel EGFL7 biosynthesis and miRNA-126 degradation, as also discussed further below. Future studies employing more and highly specific anti-EGFL-7 antibodies will be required to address this important question.

Estimates of EGFL7 VA in primary tumours of stages II and III were significantly higher in the case of relapsing disease and primarily disseminated tumours (stage IV), compared to tumours without relapse. These results indicate a possible role of EGFL7 in the metastatic process of CRC cells at an early disease stage. This is in accordance with EGFL7 functioning as a pro-angiogenic protein enhancing tumour growth and potentially also dissemination of tumour cells [10,32,33]. We were unable to identify previous studies analysing the protein expression of EGFL7 in early stage CRC. One study, however, by Díaz *et al.* analysed the EGFL7 mRNA expression in 110 patients with CRC stage I-IV, and demonstrated a significantly higher EGFL7 mRNA expression in stage III and IV than in stage I and II tumours, which supports the present results [11]. Furthermore, pre-clinical studies have demonstrated a relationship between high EGFL7 expression and increased risk of metastatic spread in hepatocellular, breast, and lung cancer [10,12]. A similar relationship has been demonstrated by analysing EGFL7, using IHC in samples from patients with laryngeal carcinomas [13].

On the other hand, we found no significant differences in VA estimates of miRNA-126 in the primary tumours according to disease stage or relapse. Previous studies in CRC have shown divergent results indicating prognostic as well as no prognostic impact of miRNA-126 in early disease stages. Moreover, a higher as well as a lower expression of miRNA-126 in CRC tissue compared to normal colonic tissue have been reported [11,23,24,26,34]. There may be multiple explanations such as differences in the methodological approach, sample sizes, tissue used for analyses, quantification methods, normalisation procedures etc., which are all known to influence the final results [27]. Also, the multiple known targets of miRNA-126 may blur the results of the present analysis or perhaps miRNA-126 is more important (as a biomarker) in the metastatic setting than at earlier disease stages in this particular context.

A positive significant correlation was demonstrated between the VA estimates of EGFL7 and miRNA-126 in stage III and IV tumours. Since the miR-126 sequence is located within the EGFL7 mRNA precursor, some degree of correlation in expression levels would be expected, although we are here considering the EGFL7 protein and the miRNA-126 (transcript). Some investigators have found a positive correlation between EGFL7 and miR-126 [16,35-37], others have, interestingly, been unable to detect such an association [11,16], which supports the lack of significant correlation in the present study.

The VA estimates of EGFL7 were significantly higher, and the estimates of miRNA-126 significantly lower, in regional lymph node metastases than in primary tumours. This corresponds rather well with the assumption of high EGFL7 and low miRNA-126 expressions being associated with an increased metastatic potential. The correlation between VA estimates of EGFL7 and miRNA-126 in the lymph node metastases only reached borderline significance, tentatively supporting a positive correlation between the estimates. We were unable to identify previous studies assessing estimates of EGFL7 and miRNA-126 expression in paired samples of primary CRC and lymph node metastases.

In the present study VA estimates of EGFL7 and miRNA-126 were obtainable from all analysed metastatic lesions at different organ sites. Although VA estimates of miRNA-126 differed significantly between lung and peritoneal metastases, further comparisons were restricted to liver metastases for reasons of statistical power. We detected no differences between VA estimates of EGFL7 in the liver metastases and primary tumours or regional lymph node metastases. However, the VA estimates of the miRNA-126 were significantly lower in liver metastases than in primary tumours. This finding is comparable with the difference between primary tumours and regional lymph nodes, suggesting a general down-regulation of the miRNA-126 expression in metastatic lesions. This is consistent with previous reports on breast cancer, where miRNA-126 has been documented to be down-regulated in metastatic tissue compared to primary tumours [36,38,39]. A possible difference, although non-significant, was also detected between VA estimates of miRNA-126 in liver and regional lymph node metastases. These results are in line with the study by Png *et al.* in which a suppressing role of metastatic initiation and metastatic colonization was demonstrated for miRNA-126 [39]. These effects were mediated through suppression of EC recruitment to the metastatic lesions caused by suppression of the *IGFBP2*, *PITPNCI*, and *MERTK* genes. Together with the results obtained in the primary tumours, these data may indicate that EGLF7 and miRNA-126 exert their angiogenic influences differently, depending on the stage of the disease. In accordance with the above comparisons a positive significant correlation between VA estimates of EGFL7 and miRNA-126 was seen in liver metastases, suggesting a conserved regulation of their common expression at the distant metastatic organ site.

## Conclusion

The current descriptive study holds the natural limitations of a retrospective investigation and the results are hypothesis generating. However, the present design with paired samples adds information on the examined biomarkers in a clinical setting, which is rarely available in translational research. Overall, the results correspond rather well with the assumption of high EGFL7 and low miRNA-126 expressions being associated with an increased metastatic potential.

- The results argue for a potential role of EGFL7 in the early metastatic process leading to relapse after curatively intended surgery for stage II and III CRC and call for further clinical validation.
- A pronounced down-regulation of miRNA-126 in metastatic lesions compared to the primary tumour was detected supporting its role in the metastatic process.
- The present results demonstrated a significant correlation between the VA estimates of EGFL7 and miRNA-126 in advanced stages supporting a coordinated regulation of their expression.
- The visualisation of EGFL7 suggested an expression pattern with a high EC affinity, however, expression in other cell types is suggested. This observation requires further investigation.

## Additional files

Additional file 1: Figure S1. Specificity of the EGFL7 antibody. Additional file 2: Figure S2. Correlations between EGFL7 and miRNA-126. Additional file 3: Table S1. EGFL7 and miRNA-126 in distant metastases.
