# Supplementary material for: Intra-tumoural vessel area estimated by expression of epidermal growth factor-like domain 7 and microRNA-126 in primary tumours and metastases of patients with colorectal cancer: a descriptive study
Source: J Transl Med. 2015 Jan 16;13:10. doi: 10.1186/s12967-014-0359-y (PMC4302134; doi:10.1186/s12967-014-0359-y)
Supplement: Additional file 1: Figure S1. — Specificity of the EGFL7 antibody. [file 12967_2014_359_MOESM1_ESM.pdf]

Supplementary Figure 1

Testing the specificity of the EGFL-7 antibody was accomplished by an immunological pre-incubation / substitution approach. The pre-incubation of EGFL7 antibody and antigen prior to immunoperoxidase staining was performed as described in Materials and Methods.

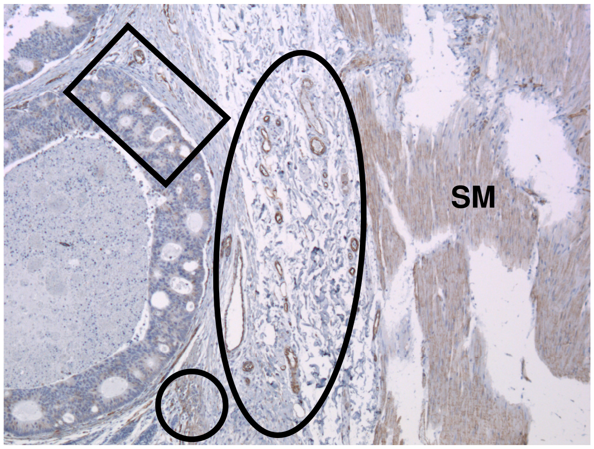

A. The invasive front of a colon adeno-carcinoma, stained with rabbit polyclonal, primary antibody to EGLF7 (ab115786, Abcam Cambridge, UK; 1:200). Strong immunoperoxidase staining (dark brown) is seen in vessels (oval frame), whereas weaker staining is seen in smooth muscle cells (SM), a few adenocarcinoma cells (square) and focal stromal cells in the invasive front of the tumour (circular frame).

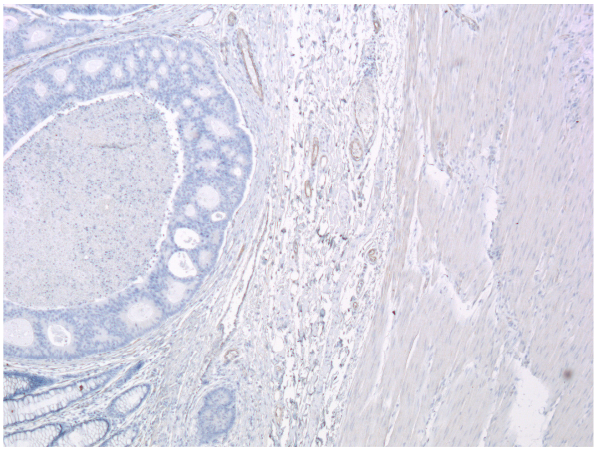

B. Tissue section, cut adjacent to A, stained with the same antibody after pre-incubation with EGFL7 recombinant protein (Novus Biologicals H00051162-P01), using approximately 2.4 μM antigen, *versus* 0.6 μM antibody (1:200). The immuno-peroxidase staining in vessels is greatly reduced and the staining in the other cellular compartments is almost abolished.
