# Supplementary material for: Intra-tumoural vessel area estimated by expression of epidermal growth factor-like domain 7 and microRNA-126 in primary tumours and metastases of patients with colorectal cancer: a descriptive study
Source: J Transl Med. 2015 Jan 16;13:10. doi: 10.1186/s12967-014-0359-y (PMC4302134; doi:10.1186/s12967-014-0359-y)
Supplement: Additional file 2: Figure S2. — Correlations between EGFL7 and miRNA-126. [file 12967_2014_359_MOESM2_ESM.pdf]

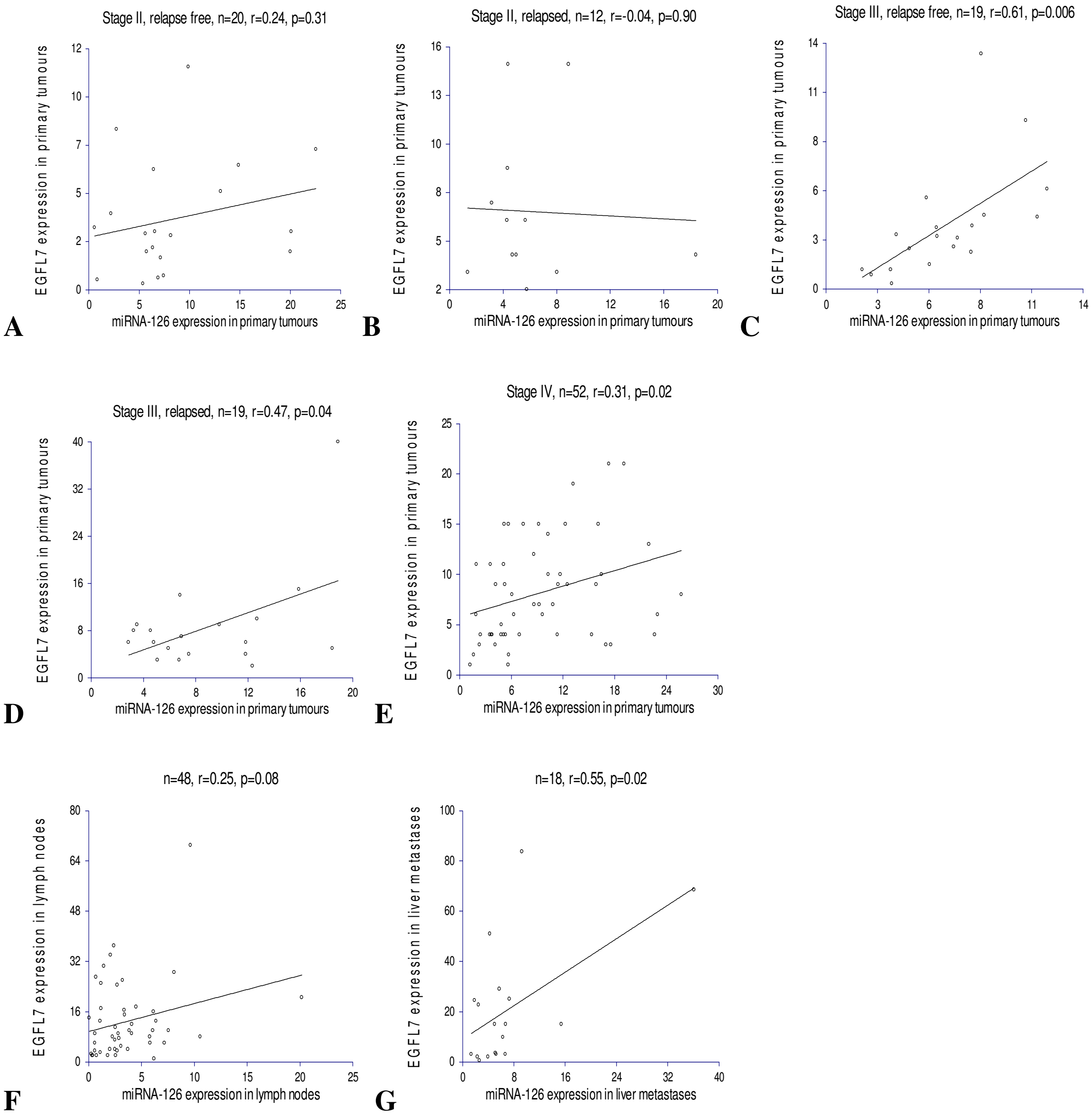

Supplementary Fig. S1 Correlations between intra-tumoral expressions of Epidermal growth factor-like domain 7 (EGFL7) and microRNA-126 (miRNA-126) assessed as vessel area fractions in **a**, primary tumours stage II, relapse free **b**, primary tumours stage II relapsed **c**, primary tumours stage III relapse free **d**, primary tumours stage III, relapsed **e**, primary tumours stage IV **f**, regional lymph nodes, and **g** liver metastases.
