# Supplementary material for: Intra-tumoural vessel area estimated by expression of epidermal growth factor-like domain 7 and microRNA-126 in primary tumours and metastases of patients with colorectal cancer: a descriptive study
Source: J Transl Med. 2015 Jan 16;13:10. doi: 10.1186/s12967-014-0359-y (PMC4302134; doi:10.1186/s12967-014-0359-y)
Supplement: Additional file 3: Table S1. — EGFL7 and miRNA-126 in distant metastases. [file 12967_2014_359_MOESM3_ESM.doc]

**Supplementary Table 1** Estimates of epidermal growth factor-like domain 7 (EGFL7) and microRNA-126 (miRNA-126) vessel area fraction according to the location of distant metastases

| **Location of metastases** | **EGFL7** vessel area | | **miRNA-126** vessel area | |
| --- | --- | --- | --- | --- |
|  | Number | Median | Number | Median |
| Liver | 18 | 15.0 | 20 | 5.2 |
| Lung | 5 | 18.0 | 7 | 2.4 |
| Peritoneal | 3 | 26.0 | 4 | 9.5 |
| Omental | 2 | 15.5 | 2 | 3.3 |
| Abdominal | 0 |  | 1 | 7.7 |
| Small intestine | 0 |  | 1 | 6.1 |
| Distant lymph nodes | 2 | 30.5 | 2 | 8.5 |
| Estimates of microRNA-126 vessel area fractions in lung metastases was significantly lower than in peritoneal metastases (p=0.047). For the other metastatic sites there was no significant difference between estimates of vessel area fractions regarding both EGFL7 and miRNA-126. | | | | |
